# Society Meetings

**Published:** 1905-03

**Authors:** 


### SOCIETY MEETINGS.

The Homeopathic Medical Society of the State of New York held its annual meeting at Albany, February 14 and 15, 1905, at which the following-named officers were elected: president, Dewitt G. Wilcox, Buffalo; vice-presidents, W. T. Helmuth, New York, B. W. Sherwood, Syracuse, George R. Critchlow, Buffalo; secretary, H. Worthington Page, New York; treasurer, C. T. Haines, Utica.

THE Esculapian Club held its regular monthly meeting at the Hotel Touraine, Thursday evening, February 16, with Dr. Julius Ullman as host. Dr. F. S. Hoffman presented a paper on Nephrolithiasis.

---

THE Buffalo Academy of Medicine held meetings during the month of February, 1905, as follows:

*Section on Surgery.*—Tuesday evening, February 7. Program: (*a*) Time as an element in abdominal surgery, Maurice H. Richardson, Boston; (*b*) Classic art in medicine and surgery, lantern exhibit, Roswell Park.

*Section on Medicine.*—Tuesday evening, February 14. Program: The need of a medical library in Buffalo, William C. Krauss, (by request).

---

THE American Anti-Tuberculosis League will hold its next annual meeting at Atlanta, Ga., April 17-19, 1905, under the presidency of Dr. George Brown, of Atlanta, who is the executive officer and to whom all communications should be addressed.

